# Grounding the Selectionist Explanation for the Success of Science in the External Physical World

**DOI:** 10.1007/s10699-023-09907-y

**Published:** 2023-03-13

**Authors:** Ragnar van der Merwe

**Affiliations:** University of Johannesburg, Johannesburg, Gauteng South Africa

**Keywords:** Bas van Fraassen, K. Brad Wray, Scientific realism, Scientific anti-realism, Scientific explanation, Scientific revolutions

## Abstract

I identify two versions of the scientific anti-realist’s selectionist explanation for the success of science: Bas van Fraassen’s original and K. Brad Wray’s newer interpretation. In Wray’s version, psycho-social factors internal to the scientific community – viz. scientists’ interests, goals, and preferences – explain the theory-selection practices that explain theory-success. I argue that, if Wray’s version were correct, then science should resemble art. In art, the artwork-selection practices that explain artwork-success appear faddish. They are prone to radical change over time. Theory-selection practices that explain theory-success in science are however not faddish. They are mostly stable; that is, long-lived and consistent over time. This is because scientists (explicitly or implicitly) subscribe to what I will call the testability norm: scientific theories must make falsifiable claims about the external physical world. The testability norm and not psycho-sociology explains the theory-selection practices that explain theory-success in science. Contra Wray, scientific anti-realists can then maintain that the external physical world (as expressed in the testability norm) explains theory-success.

**Supplementary Information:**

The online version contains supplementary material available at 10.1007/s10699-023-09907-y.

The struggle for existence holds as much in the intellectual as in the physical world. A theory is a species of thinking, and its right to exist is coextensive with its power of resisting extinction by its rivals.

– T. H. Huxley (1893, p. 319).

## Introduction

How should we explain the success of science? This is an important question about which there is ongoing disagreement. Several scientific anti-realists – notably K. Brad Wray – maintain that Bas van Fraassen’s (1980) *selectionist explanation for the success of science* (SESS) provides the answer. The success of science is understood metaphorically in terms of the survival of certain scientific theories: those theories that have outcompeted rival theories.^1^ Successful theories are those that have been *selected* rather than those that are tracking truth or unobservables (as realists often suppose). The survival of scientific theories is analogous^2^ to the survival of biological organisms, and scientists *choosing* between theories is analogous to natural selection ‘choosing’ between organisms.

My aim in this paper is to argue that Wray’s version of SESS overemphasises the degree to which psychological and social factors explain the theory-selection practices that explain theory-success in science. Psychological factors include scientists’ interests, goals, and preferences. Social factors include communal standards and consensuses among scientists. I will refer to these jointly as *psycho-social factors*. Broadly, a factor is any entity, structure, state, or event that can compel, inform, or be taken into consideration while securing norms for theory-selection in science. I argue that, were psycho-social factors the primary explanatia of theory-selection, then science should resemble art. In art, the artwork-selection practices that explain artwork-success appear faddish. They are prone to radical change over time. The theory-selection practices that explain theory-success in science are instead relatively stable. They are long-lived and consistent over time. I argue that this is because scientists are (explicitly or implicitly) subscribing to what I will call the *testability norm.* The testability norm stipulates that scientific theories must make falsifiable claims about the external physical world.

By ‘falsifiable’, I mean that scientific theories must, in principle, be falsifiable by empirical means. This is not only the case in physics. Even while there has been debate over whether evolutionary theory constitutes a genuine scientific theory (e.g. Popper, 1978; Fodor and Piattelli-Palmarini 2010), its central claims are still empirically testable (e.g. by being supported in the fossil record or in data generated by DNA sequencing). This must be the case for evolutionary theory to be a science rather than a kind of pseudo-science or metaphysics (Boudry et al., 2015). That said, talk of falsifiability does not require subscription to Popper’s scientific methodology, nor to a ‘logic of scientific discovery’ (or ‘logic of theory-success’ in our case). Falsifiability should instead be understood in the broad sense that scientists seem to use the term: as connoting as insistence that scientific theories be testable against the external physical world in some or other way. Novel prediction alone is a not enough since there is no way to confirm whether a prediction is successful without making empirical contact with the external physical world (see Fahrbach, 2011, 2017; van der Merwe, forthcoming-a). By ‘external physical world’, I mean the non-abstract, non-institutional, non-social, and non-psychological ontological domain ‘out there’ that constitutes the subject matter of the natural sciences.

My positive argument can be syllogised as follows:

P1: As per SESS, scientists’ selection practices explain theory-success.

P2: The external physical world explains scientists’ selection practices.

C: Therefore, the external physical world explains theory-success.

The external physical world is thus the *ultimate*, rather than the *proximate*, explanation for theory-success (see Mayr, 1963 and Scott-Philips et al., 2011 for more on ultimate versus proximate explanations). Contra Wray, even scientific anti-realists who subscribe to SESS can then claim that the external physical world explains theory-selection and therefore theory-success.

In Sect. 2 of this paper, I outline Wray’s version of SESS and how it differs from van Fraassen’s original. Despite Wray’s professed allegiance to van Fraassen’s Constructive Empiricism, his view deviates significantly therefrom.

In Sect. 3, I argue contra Wray’s SESS that the testability norm is the primary explanans in theory-selection (I suggest that this is also van Fraassen’s view).

In Sect. 4, I engage with two possible counter arguments: (1) Kuhn has shown that scientific norms undergo radical upheaval during scientific revolutions; (2) Feyerabend has shown that science and art are analogous in that both permit a plurality of contingent representational strategies. In response, I argue that, although Kuhn and Feyerabend did claim as much, they did not question the centrality of what I am calling the testability norm.

Note that my aim is not to defend either scientific realism or anti-realism. I will therefore not engage with the standard arguments in the realism/anti-realism debate (e.g. the no-miracles argument, the pessimistic meta-induction, and the underdetermination argument). Instead, my argument is specifically focused on SESS. It takes place within that general framework of suppositions and commitments. I will therefore not defend SESS against realist attacks (see however Wray, 2019, chs. 9–11 and Lee, 2021 for an overview of that debate). I am also not assuming the correctness of van Fraassen’s SESS, and then defending it against Wray’s version. I contrast Wray’s SESS with van Fraassen’s SESS because Wray is concerned with van Fraassen’s version. In attempting to explain the success of science, both realists and van Fraassen assign explanatory primacy to the external physical world, while Wray assigns explanatory primacy to psycho-social factors. My argument should therefore make a novel contribution to the debate. It has not been argued that SESS can fail on anti-realism’s own terms. Neither, to my knowledge, has there been detailed discussion of the respectively stable versus faddish natures of selection practices in science versus art.

Some may object up front that it is an empirical matter whether internal psycho-social factors or external worldly factors explain theory-success in science. We can simply ask scientists what explains their theory-selection practices (Mizrahi, 2020 takes such an x-phi approach). This may be informative, but what scientists *say* might not truly reflect what norms are *de facto* operant in science. Furthermore, my concern is specifically with what *explains* the theory-selection that *explains* theory-success. And, explanation requires philosophical work (even if undertaken in conjunction with empirical studies).

Lastly, note that I will sometimes speak of ‘grounding’, ‘determination’, and ‘mechanism’. These terms are not intended to carry metaphysical baggage. Talk of ‘mechanism’, for example, does not entail a commitment to either mechanistic explanations or to mechanisms as metaphysically robust entities. Instead, ‘mechanism’ merely denotes a locus of explanatory primacy.

## ESS: A Metaphorical Explanation for Theory-Success

The purpose of this section is to outline SESS as presented by van Fraassen and Wray (see also van der Merwe 2020). I will discuss the similarities between their views, but also emphasise a key difference. This is that van Fraassen remains an empiricist about SESS, while Wray shifts SESS’s explanatory focus to psycho-sociology.^3^

### What Motivates SESS?

Scientific anti-realists like Wray argue that realists cannot sustain commitment to the truth, approximate truth, or convergence to truth of our best scientific theories.^4^ Nor can realists claim that our best scientific theories track mind-independent unobservables (e.g. natural laws or fundamental entities). This is because we can infer that the radical Kuhnian change identifiable in the history of science will continue. Even our best current theories will most likely be discarded and replaced in the future.

Realists often respond that the success of science would be a *miracle* if scientific theories where not (at least, approximately) true, and if they did not track some mind-independent reality populated by unobservables. For van Fraassen, there is however no need for such an invocation of miracles to explain the success of science. Scientific practice, he says, consists in a three-way interaction between scientist, theory, and *observable* phenomena. The function of scientific theories is to account of what is *actual*. There may or may not be an explanation “in terms of unobservable facts ‘behind the phenomenon’ – it really doesn’t matter to the goodness of the theory, nor to our understanding of the world” (van Fraassen 1980, p. 24). We should instead think of science as an evolutionary process in which only successful theories survive. Van Fraassen invokes an analogy related the predator-prey relationship between cats and mice. The Darwinist, he says, does not ask “why the mouse runs from its enemy. Species which did not cope with their natural enemies no longer exist. That is why there are only ones who do” (van Fraassen, 1980, p. 39; see also Wray. 2007, pp. 84–87). Likewise, the anti-realist about theory-success does not ask why some theories *qua* theories survive. Instead, she simply accepts that those which were unsuccessful were not selected and are therefore not around anymore.

Although van Fraassen only devotes two paragraphs in *The Scientific Image* to SESS (1980, pp. 39–40), Wray has expanded the idea into a genuine anti-realist candidate for explaining theory-success (see notably 2007, 2010a, 2013: 1724–1725, 2018 chs. 9 and 10). SESS, says Wray, is not about why some specific scientific theory is successful or about identifying some common feature amongst successful theories. It is instead an attempt to explain why we have successful theories at all (Wray, 2007, p. 83, 2010a, pp. 367 − 274, 2020; van Fraassen, 1980, p. 219, fn. 34; see also Boyce, 2018 and Lee, 2021).^5^ The anti-realist selectionist explains theory-success in terms of “a single mechanism, a selection mechanism” (Wray, 2010a, p. 375). This focus on selection as the single mechanism responsible for theory-success places a large emphasis on the role of scientists (*qua* selectors). Wray recognises that

the predictive success of our current best theories is a consequence of the fact that theories that do not save the phenomena, that is, theories that fail to account for what has been observed, tend to be discarded (2018, p. 174).

Nonetheless, Wray maintains that predictive success is determined by the currently accepted standards of success, and these standards are determined by psycho-social factors such as scientists’ contingent research interests, values, goals, and preferences. Saving the phenomena or predictive success are only some of several factors scientists might (or might not) consider during theory-selection depending in their interests, values etc. On Wray’s account, it is these psycho-social factors – interests, values etc. – that ultimately explain theory-selection and therefore theory-success (Wray, 2010a, pp. 369–375, 2013, pp. 1724–1725, 2019, pp. 560–564).^6^ Psycho-social factors explain what counts as saving the phenomena and predictive success in the first place. Even if saving the phenomena or predictive success can be proximate explanations for theory-selection, psycho-social factors are the ultimate explanation (recall the introduction). As Wray puts it,

[s]cientific inquiry is interest-driven. Which specific features a theory is designed to account for, as well as which specific features it disregards or brackets, is *determined by* the research interests of scientists. Theories are developed with specific research problems and goals in mind (2018, p. 190 emphasis added).

While there is, of course, some truth to this claim, note that Wray does not stress the role of community-extrinsic empirical phenomena (as a van Fraassian empiricist would). Instead, he thinks that theory-design is “determined by” scientists’ community-intrinsic interests, viz. psycho-social factors.^7^

An anonymous reviewer argued that Wray is only committed to the following claim:

C1: Community-*intrinsic* factors are necessary for the determination of scientific norms.

According to the reviewer, Wray is not committed to the following claim:

C2: Community-*extrinsic* factors are not necessary for the determination of scientific norms.

Endorsing C1 does not entail endorsing C2. As the reviewer pointed out, endorsing that condition type X is necessary for Y does not entail endorsing that condition non-type X is not necessary for Y. This last statement is, of course, correct. However, there is textual evidence to suggest that Wray is, in fact, committed to C2. We are though only concerned with norms related to theory-success since this is what SESS is about.

Wray states that which theories “count as successful is determined, to a large extent, by what scientists are prepared to tolerate and what they have to choose from” (Wray, 2018, p. 168). Thus, although saving the phenomena or predictive success can play a role, Wray does not consider them to be key to an explanation of theory-success. “Strictly speaking, even less is required. All a theory needs to do is be more successful than existing competing theories” (Wray, 2017, p. 45). Furthermore, “given that scientists set their own standards of success, it should not surprise us that our current theories are successful” (Wray, 2018, p. 165; see also 2010a, 2019). These quotes suggest that Wray (perhaps tacitly) subscribes to C2. If a theory only needs to be more successful than its rivals, and, if the scientific community decides what counts as success, then community-extrinsic factors are not necessary for the determination of scientific norms. Wray seems to be saying that the scientific community could, in principle, arbitrarily decide which rules apply. Members of the scientific community’s research interests are not bound by community-extrinsic factors if scientists can “set their own standards of success”.

Wray further states that “provided there is some significant shift in research interests in a research community, a theory that seemed adequate at one time may come to seem unacceptable later” (Wray, 2018, p. 194). Again, this suggests that Wray is committed to C2. He does not say that research interests *bound by empirical evidence* determine theory-success. Rather, he seems to think that research interests *simpliciter* determine theory-success. Research interests (viz. community-intrinsic factors) can then, in principle, come apart from empirical evidence and predictive success (viz. community-extrinsic factors). Moreover, norms for theory-selection are largely

the result of social consensus in the research community. That is, the degree of accuracy that is deemed acceptable is determined by what one’s fellow researchers accept as accurate (Wray 2018, p. 165; see also 2019).

Importantly, scientists “can change the standards to ensure that at least some theory passes through” (Wray, 2018, p. 165). Here, we can again see Wray’s commitment to C2. A theory need to not conform to the evidence or be predictively successful. Instead, what “passes through” – what is successful – is decided by the scientific community. And, the scientific community may or may not take community-extrinsic factors into account. On Wray’s account, research interests decide scientific norms. As before, research interests are not bound by community-extrinsic factors if scientists “can change the standards to ensure that at least some theory passes through”.

The above makes it clear that Wray’s SESS renders empirical and predictive success secondary to psycho-social factors. The latter is necessary while the former is not necessary for theory-success. In principle, theories can be false in – not only their alethic and ontological, but also their empirical consequences – yet still survive the process of competition and selection. Psycho-social factors may align with empirical considerations in theory-selection, but they need not do so for theory-success to obtain. On Wray’s account, psycho-social factors thus have explanatory primacy over empirical factors when it comes to theory-selection (see also Psillos, 2020).

What it means for psycho-social factors to have explanatory primacy over empirical factors is as follows. For Wray, scientists’ interests, goals, and preferences determine if, when, why, and how empirical factors contribute to theory-selection, and not the other way around. In other words, empirical factors can (partly) explain theory-selection, but psycho-social factors explain *both* empirical factors and theory-selection. It in this sense that Wray’s SESS grants explanatory primary to psycho-social factors over empirical factors. Both empirical-factors and theory-selection are explained by psycho-sociology, while empirical factors can, at best, sometimes partly explain theory-selection. Empirical factors are explanatorily constrained or bounded by psycho-social factors in a way that is not reciprocated.

We can think of community-intrinsic factors (interests, goals etc.) as *primary* in Wray’s SESS and community-extrinsic factors (e.g. predictive power or empirical evidence) as *secondary*. A primary factor is a factor (as defined in the introduction) that is *determining* of some pertinent norm. A primary factor is *necessary* for the norm to obtain. It constrains, binds, or compels – it ‘forces the hand’ of – those deciding, subscribing to, and articulating the norm. A secondary factor is a factor that can, but need not, play this role. It is neither determining nor necessary. It might play an informative role – it might be taken into consideration or utilized – but it does not force the hand of those deciding, subscribing to, and articulating the norm.

In Wray’s SESS, the determination of scientific norms can occur without community-extrinsic factors playing any determining role. My counterargument (Sect. 3) is that, for science to be science (rather than like art), scientists are constrained by – their norms are determined by – empirical factors rather than psycho-social factors. Contra Wray, the former are primary and the latter are secondary.

Although analogies are never perfect, consider a court of law that must decide whether some defendant is guilty of murder. The court will consider various factors. Circumstantial evidence or eyewitness reports, for example, will be what I have called secondary factors. In contrast, empirical forensic evidence – DNA traces or fingerprints, for example – can be thought of as primary factors. The primary factors, in a sense, *determine* the court’s verdict; they ‘force the hand’ of the court. Given how the legal system works (in democratic, free societies), the court must – it is *necessary* that the court – base its verdict on primary factors (when available). In contrast, the court might only consider or let its decision be informed by secondary factors. All things being equal, secondary factors alone are not determinant or necessary in the way that primary factors can be. If the court is not operating this way, then something has gone wrong. It would not be following proper legal procedures. It would be a deficient court (perhaps a corrupt or incompetent one).

In this analogy, primary factors are determining, binding, or necessary for a suitable outcome even if secondary factors can play a subsidiary role. Now, of course, there are cases where primary factors are not available or where they are derived using unreliable methods. Secondary factors may then come to the fore. Nonetheless, a court of law should, in principle, work roughly the way I have described. If not, then law *qua* law is not being properly practiced. My claim (to be fleshed out in Sect. 3) is that something similar is going on in science.

### Internalist SESS Versus Externalist SESS

Importantly, Wray diverges from van Fraassen when he underplays the role of observable phenomena in his SESS. In an oft-quoted phrase, van Fraassen states that

any scientific theory is born into a life of fierce competition, a jungle red in tooth and claw. Only the successful theories survive – the ones which *in fact* latched on to actual regularities in nature (van Fraassen, 1980, p. 40 original emphasis).

Here, van Fraassen seems to suggest that successful theories have the property of latching onto actual regularities in nature. As noted, SESS is however not about identifying some common feature amongst successful theories. It is instead an attempt to explain theory-success in terms of a selection mechanism. Van Fraassen does not discuss whether empirical versus psycho-social factors are necessary for theory-selection. As a committed empiricist, he would though presumably grant empirical factors explanatory primacy over psycho-social factors. If so, then the selection and resultant success of theories will be determined by empirical rather than psycho-social factors.

Even if van Fraassen were to reject my suggestion that he appeals to empirical factors in explaining theory-selection, there does not appear to be any principled reason why a generic empiricist could not do so. I therefore take it that this option is – at least in principle – open to van Fraassen. However, it does not appear to be open to Wray and those who defend similar psychologically or sociologically oriented explanations for theory-success. Although Wray agrees that successful theories are referencing observable phenomena in some way, he does not seem to think that observable phenomena play a determinant role in SESS. As noted, he emphasizes the role of interests, goals etc. Wray is mostly concerned with the two-way interaction between scientist and theory in contrast to van Fraassen’s three-way interaction between scientist, theory, *and* observable phenomena (Rowbottom, 2019 and Vickers, 2020 make similar points). The explanatory locus in Wray’s SESS is in psycho-sociology, while the explanatory locus in van Fraassen’s SESS is in the external physical world, viz. observable phenomena.

Despite outwardly subscribing to van Fraassen’s Constructive Empiricism, Wray subtly shifts SESS from what was originally an *externalist* to an *internalist* explanation for theory-success (Bloor et al., 1996 and Longino, 1990 develop similar views). As Steven French notes, “[t]his is crucial to Wray’s overall argument: theories are replaced because they are inadequate but what makes them so is that researchers’ interests have changed” (2020, p. 6). Like me, French is concerned that Wray’s emphasis on research interests and the like overlooks the role of empirical successes and failures in theory-change. In a sense, van Fraassen’s externalist SESS is then closer to the realist’s explanation for theory-success than to Wray’s internalist SESS. Both van Fraassen and the realist can appeal to some explanans resident in the external physical world: observable and unobservable phenomena respectively.

Given the above, there seem to be two versions of SESS: an internalist conception (in terms of psycho-social, community-intrinsic factors) and an externalist conception (whether in terms of observable or unobservable community-extrinsic factors). Let us call these two views SESS_int_ and SESS_ext_ respectively. Wray and likeminded psycho-socially oriented thinkers reside in the SESS_int_ camp, while realists and Constructive Empiricists reside in the SESS_ext_ camp. Note however that both SESS_int_ and SESS_ext_ recognize that internal *and* external factors can be mutually engaged in theory-selection. The difference is that the former grants explanatory primacy to that which is internal to the psychological and social workings of science, while the latter grants explanatory primacy to that which is external to the psychological and social workings of science, viz. empirical interactions with the external physical world.

An anonymous reviewer was concerned that I have erected a straw man here. Since Wray explicitly states that he subscribes to van Fraassen’s Constructive Empiricism, I must be mistaken in portraying him as granting primacy to psycho-social factors over empirical factors. Clearly, Wray wants to be an empiricist, and he does, at times, express empiricist views. My claim is not that he explicitly professes to a non-empiricist view. Instead, my claim is that his repeated emphasis on psycho-social factors and the (often tacit) consequences of his view render empirical factors secondary to psycho-social factors. I do not see how a careful reading of Wray’s topical writings can lead to any other conclusion. Much of Wray’s writings is taken up with arguments that stress the role of choices, judgements, interests, values, etc. in science, and he largely underplays empirical concerns. If he really believes that empirical factors are primary over psycho-social factors, then why does he repeatedly emphasise how empirical factors are subsidiary to or a product of psycho-social factors?

Even when Wray takes empirical factors into consideration, what counts as an empirical factor in the first place is determined by psycho-social factors (see also Psillos 2020 and Vickers 2020). On my reading, Wray’s view thus appears closer to Barnes and Bloor’s Strong Programme (e.g. Bloor et al. 1996) than to van Fraassen’s Constructive Empiricism. If what counts as empirical success is determined by research interests, community standards, and the like rather than by empirical phenomena, then we are not dealing with a strictly van Fraassian kind of empiricism (and perhaps not any kind of empiricism) anymore.

I now argue that Wray’s SESS_int_ does not adequately explain theory-selection and therefore theory-success in science.

## Against SESS_int_: The Testability Norm and the Stability of Science

In this section, I argue that theory-selection is explained by what I will call the *testability norm* rather than by psycho-social factors. The testability norm:

Scientific theories should, in the main, be selected according to considerations of the degree to which they make claims about the physical world that are falsifiable by empirical means, notably observation^9^ and novel prediction.

An obvious objection at this point – one that Wray would surely make – is that the testability norm is itself determined and therefore explained by psycho-social factors (Bloor, 1976 and Ambrosio, 2021 argue along similar lines). An anonymous reviewer suggested that it is trivially the case that the scientific community decides its own norms. My argument is precisely that this is not the case. If it were, science would not be science. The scientific community is not free to posit and subscribe to whatever norms they fancy (as SESS_int_ seems to suggest). My argument is that the scientific community is bound or constrained (their ‘hand is forced’) by community extrinsic factors, viz. empirical concerns with the external physical world. Were this not the case, then scientists would not be practicing science, and science would be faddish in the way that art is.^10^

Now, norms are admittedly advanced and held by persons or communities of persons. So, yes, there is a sense in which psycho-social factors *inform* the testability norm. However, psycho-social factors do not *determine* the testability norm. Psycho-social factors may *determine* other norms for theory-selection, but there is a crucial difference between the testability norm and other norms for theory-selection. Other norms include impartiality, replicability of results, peer review standards, and considerations of so-called theoretical virtues (e.g. logical consistency, semantic coherence, simplicity, and unificatory and explanatory scope). The difference, I believe, is that scientists cannot – must not – abandon the testability norm. The testability norm is *binding* on the scientific community in a way that other norms are not.

For scientists to be doing science *qua* science, they must place the empirical considerations entailed in the testability norm front-and-centre. Other norms for theory-selection do not enjoy this primacy. Considerations of theoretical virtues, for example, only play a significant role when the testability norm cannot decide between empirically equivalent theories; that is, in cases of underdetermination. As Sandra Mitchell notes, “empirical evidence continues to serve as a methodological foundation for the acceptance or revision of scientific beliefs” (Mitchell, 2020, p. 184; see also Sarton, 1963; Lakatos, 1978; Fahrbach, 2011, 2017; Mizrahi, 2020, Psillos, 2020; van der Merwe, forthcoming-a, forthcoming-b). Alternatively, “[e]mpirical test remains the arbiter of scientific worth” (Mitchell, 2009, p. 108); and empirical testing, by definition, involves some reference to and interaction with the external physical world.

Testability is arguably definitive of scientific inquiry (of which theory-selection is a part). The testability norm is a special kind of norm that is qualitatively different from the other norms for theory-selection. It is a necessary – if not sufficient – condition for theory-selection, while other norms for theory-selection are neither necessary nor sufficient. Scientists are not free to side-line the testability-norm in the way that they might do with the other norms. It follows that the testability norm is largely determinant of – it is the primary explanans for – scientists’ interests, goals, and preferences rather than the other way around (I expand on the claims in this paragraph in Sect. 4.2).

Wray does not distinguish between different kinds of norms that may influence theory-selection. As mentioned, he thinks that psycho-social factors determine – i.e. have explanatory primary over other factors in – theory-selection. Wray uses Newton as an example:

Given a different set of research interests, scientists would be led to account for different features than those they accounted for. For example, [Newton’s] research interests, being different from those of his predecessors, dictated a change in the sorts of things he sought to account for (2018, p. 191).

Wray thus seems to suggest that the Newtonian revolution occurred because Newton’s research interests were different from those of his predecessors. It is however not at all clear that this is the case. As Stathis Psillos (in his critique of Wray’s 2018) notes, the

Cartesian (and Leibnizian) vortex theories were abandoned not because Newton’s research interests had shifted, but because they were shown to be incompatible with the *evidence* of the motion of the planets (2020, p. 21 emphasis added).

If Psillos is correct, then something like the testability norm, rather than Wrayian psycho-social factors, explains the theory-selection practices that ushered in the Newtonian paradigm.

Although often stressing the sociological character of science, David Hull likewise states that for science “to count as science, testing must… be possible. More than that, tests must be carried out and the results taken seriously” (2001, p. 352). John Maynard Smith (commenting on Hull), likewise, states that the “essential difference” between the functioning of scientific communities and the functioning of religious, political, or artistic communities is “of course… that ideas in science are subject to experimental test, whereas those in other fields are not” (1988, p. 1182; see also Bernal 1971; Toulmin, 1972; Schindler, 2018, ch. 1). Science – like art (and other human enterprises) – of course grows within a social network. Science is however unique in that scientists’ claims must be falsifiable against the external physical world.

Note however that the testability norm does not require that we follow the logical positivists in trying to separate scientific theories into theoretical sentences and observation sentences, with the latter being empirically verifiable. Instead, following Popper’s evolutionary analogy for scientific change, theory-selection is

certainly not due to anything like an experimental justification of the statements composing the theory; it is not due to a logical reduction of the theory to experience. We choose the theory which best holds its own in competition with other theories; the one which, by natural selection, proves itself the fittest to survive. This will be the one which not only has hitherto stood up to the severest *tests*, but the one which is also *testable* in the most rigorous way (1968, p. 108 emphases added; see also Richard, 1987; Schindler, 2018 ch. 1).

When Popper talks of “tests” and “testable”, he is, of course, referring to empirical testing. Scientists’ consideration of and commitment to empirical testing embodies the testability norm.

I now press the point that the testability norm is the primary determinant of theory-selection. Theory-selection in science cannot be primarily determined by SESS_int_-style internalist factors because, if it were, theory-selection and therefore theory-success should be faddish. Artwork-selection in art is faddish because of the internalist nature of norms for artwork-selection. Conversely, theory-selection practices in science are mostly *stable* – i.e. long-lived and consistent over time – because of the externalist nature of norms for theory-selection.

### The Faddish Nature of Artwork-Selection

Standards and norms in art^11^ come-and-go or fade away only to return in retro guise. Pre-modern, or so-called traditional mimetic or aesthetic norms, have, at times, been replaced by deliberately and radically anti-mimetic or anti-aesthetic norms. These reactionary trends have sometimes then been replaced by a return to mimesis in the form of hyper-realist art or a return to aestheticism in the form of kitsch or twee art (see Carroll, 1993; Danto, 1997; Walton, 2007 for more on the idiosyncratic history of art). One may be tempted to say that ‘anything goes’ when it comes to artwork-selection and therefore artwork-success. This is because there is nothing resembling the testability norm operant in the artworld. Norms for artwork-selection are primarily determined internally by the art community rather than constrained by anything external such as mimetic fidelity. According to Larry Shiner (2001), art must be understood as a historical and cultural artifact that is regulated by the structural interplay of concepts, institutions, and society rather than anything to do with the external physical world (see also Dickie, 1984; Carroll, 1993; Kraut, 2007). Psycho-social factors – notably the interests, goals, and preferences of the art community – primarily determine artwork-selection.

Norms for artwork-selection taken to anti-traditionalist extremes are exemplified in Marcel Duchamp’s *readymades* (where everyday objects, like a shovel or a bottle rack, are displayed as-is) and John Cage’s *4’33”* (a ‘piece of music’ that consists of 4 minutes and 33 seconds of silence). Despite these ‘artworks’ total disregard for traditional artistic norms, they continue to be, not only ‘displayed’ and ‘performed’, but also classified and celebrated as iconic artworks in art textbooks. They are *successful* artworks.

Given the above, there appears to be no significant externalist criteria, and perhaps no stable criteria at all, constraining norms for artwork-selection in art (see Gombrich, 1950; Sarton, 1963; Kuhn, 1977a; Thompson, 2007; Uidhir and Magnus, 2011; Elgin, 2020). This is reminiscent of Wray’s conception of scientific inquiry as primarily consisting in an interaction between scientists and theories rather than scientists, theories, *and* observable phenomena (Sect. 2). In both Shiner’s art and Wray’s science, the relevant institution functions primarily via the interaction of subjects and signifiers; the signified plays no determining definitional or normative role.

Noel Carroll, interestingly, thinks of art in terms of an evolutionary analogy. Art, he says, “mutates and evolves historically… Indeed, art often mutates radically” (Carroll, 1993, p. 316; see also Gombrich, 1950; Kuhn, 1977a). As we have seen, the mechanism for this evolution in art is however internal to the workings of the artworld. The result is a faddish and largely unconstrained enterprise where oddities like Duchamp’s *readymades* and Cage’s *4’33”* have achieved iconic status. They have survived over competing artworks that subscribe to traditional norms. Such unconventional, yet successful artworks, in fact, sometimes become iconic for the very reason that they disregard traditions. This is not what we witness in science, or so I now argue.

### The Stable Nature of Theory-Selection

Some may naturally wonder why a community-extrinsic constraint on norms for theory-selection must be the physical world. Could it not be *rationality* or *truth*, for example? Scientific realists often claim as much, and it is *prima facie* plausible that rationality and truth are (at least partly) involved in theory-selection. As noted in the introduction, my aim is not to engage in the broader scientific realism debate. It does nonetheless appear that what makes science science is the key notion of empirical testing. Metaphysics and mathematics, for example, may be perfectly rational or truth-like, but they are not sciences because they do not subscribe to the testability norm (see also van der Merwe, forthcoming-a).

In any event, were science like art – if scientists’ interests, goals, and preferences determined theory-selection – we should see some rather odd theories rise to prominence in science just as some rather odd artworks rise to prominence in art. Theory-success should be whimsical and faddish if the norms governing theory-selection were not constrained by some external factor. However, scientific theories that are not aligned with the testability norm are likely to be dismissed as pseudoscience rather than elevated to iconic status, as sometimes happens in art.

Arguing against sociology-heavy interpretations of the SESS_int_ sort, Stephen Toulmin notes that this

approach to the study of scientific development… is subject to a certain self-limitation. It gives an account of scientific development in which factors *outside* the disciplinary procedures of the natural science in question are referred to only marginally, if at all. To use a biological metaphor: it studies the ontogeny or morphogenesis of a science in isolation from its ecological environment (2009, p. 179 original emphasis; see also Richards, 1987, appendix 1).

Toulmin however thinks that various political and economic factors that may influence scientific inquiry are external to the inner workings of science. Engaging with the debate over the role of politics and economics in science is outside the scope of this paper. Nonetheless, even while political and economic factors do surely influence scientists to varying degrees, it seems unlikely that they generally determine theory-selection. It is not at all obvious how political and economic factors might have even marginally influenced the selection of presiding theories like general relativity, plate tectonics, or the germ theory of infectious diseases (I discuss Feyerabend in Sect. 4.2). Something like the testability norm is a far more likely candidate. The influence of political and economic factors on scientists is moreover a psychological and/or sociological phenomenon. It therefore hinders, rather than helps, any attempt to adequately explain theory-selection.

If theory-selection were primarily determined by political and economic influences, then science would indeed be faddish (see Hull, 1988; Grantham, 2000). This is however not what we see. Norms for theory-selection in science have remained largely stable over the last three hundred years: since, at least, the Enlightenment. This is the case even if the content of theories may have undergone significant revision (see Sarton, 1963; Ben-David, 1971; Hull, 2001; Toulmin, 2009; French, 2020) (I discuss Kuhn in Sect. 4.2). As mentioned, the pertinent norms include impartiality, replicability of results, peer review standards, considerations of theoretical virtues, and most importantly considerations of the empirical consequences of theories, viz. the testability norm.

According to Ludwig Fahrbach (2011, 2017), scientific norms have, in fact, become increasingly refined and stable over time. And, there is no reason to suppose that the testability norm will be discarded any time soon. If it were, science would cease to be science. It would become a kind of metaphysics or pure mathematics detached from empirical concerns.^12^ In contrast, there have been radical shifts in artwork-selection norms during the same time-period. As intimated, it is difficult to identify anything resembling a consistent, stable, and constraining set of norms around artwork-selection practices.

## Possible Objections

I now engage with two possible objections that those inclined towards SESS_int_ may raise:

1. Kuhn has shown how science is susceptible to radical upheavals, and science therefore resembles art.
2. Feyerabend has shown that science and art are analogous in that both permit a plurality of legitimate representational strategies.

In response, I show that both Kuhn and Feyerabend, at times, advanced views consistent with the idea that the testability norm is primary in explaining theory-success in science.

### Kuhnian Revolutions

Although Kuhn warned that the analogy between biological evolution and scientific change can “easily be pushed too far”, he did consider it appropriate for explaining what he called the “resolution of revolutions”:

The process [of] resolution of revolutions is the selection by conflict within the scientific community of the fittest way to practice future science… And the entire process may have occurred, as we now suppose biological evolution did, without benefit of a set goal, a permanent fixed scientific truth, of which each stage in the development of scientific knowledge is a better exemplar (1970, pp. 172–173).

In this brief endorsement of the analogy between biological evolution and scientific change, Kuhn does not seem to think that revolutions signal a clean break between pre- and post-revolutionary paradigms (as e.g. Foucault, 1994 does). If pre- and post-revolutionary paradigms were *incommensurate* – understood as conceptually dissociated due to revolutionary upheaval – then the evolutionary analogy would be inappropriate. This is because evolution involves transitions over time rather than discontinuous jumps (Toulmin, 2009; Wray, 2010b, ch. 1). Even Gould and Eldredge’s (1992) much-discussed *punctuated equilibrium* model does not advocate for discontinuity in biological evolution. Punctuated equilibrium does not, strictly speaking, contradict Darwinian gradualism as much as suggest that there are periods of stasis that morph into and out of intermittent and short-lived periods of saltation. Gould and Eldredge, in fact, consider Kuhn’s notion of scientific revolutions to be “a punctuation theory for the history of scientific ideas” (1993, p. 227).

According to Thomas Nickles, Kuhn’s view is analogous to biological evolution if “considered on the correct time scales… Examined from afar, revolutions are simply the more noteworthy episodes in the evolution of the sciences…” (2017, np). In biological evolution, events like the Cambrian explosion appear to be episodic on a geological timescale; yet, when examined

on the timescale of the biological generations of the life forms in question, the development is evolutionary – more rapid evolution than during other periods, to be sure, but still evolutionary (Nickles, 2017, np).

Thus, on a macro-scale – on a course-grained view – there appears to be continuity through both evolutionary and scientific change. On the micro-scale – on a fine-grained view – we witness what appear to be saltations or radical changes that ostensibly render pre- and post-revolutionary periods incommensurate (see also Sarton, 1963; Toulmin, 1972; Ruse, 1989). This suggests that there can be paradigmatic continuity through Kuhnian revolutions. Kuhn’s conception of scientific revolutions does not contradict my claim that science is a mostly stable enterprise, specifically if we are concerned with norms for theory-selection. As mentioned, these have changed little since the Enlightenment. The history of art, rather than science, seems to display revolutionary upheavals rendering one ‘paradigm’ genuinely incommensurate with the next (see Gombrich, 1950; Kuhn, 1977a; Pinto de Oliveira, 2017).

Even if scientific revolutions do render norms incommensurate, commitment to the value of empirical inquiry *itself* does not change amongst scientists during revolutions. Kuhn, in fact, considered what he called “accuracy” of theories – viz. degree of fit to observable phenomena – to be “the most nearly decisive” of all the norms for theory-selection (1977b, p. 323; see also Hull, 1988; Schindler, 2018). Thus, even while the other norms listed in Sect. 3 can change (even radically), what appears to remain constant is a steadfast commitment to what I have called the testability norm.

### Feyerabend’s Analogy Between Science And art

Feyerabend (notably 1984) argued that science and art are analogous in that they both permit a plurality of representational strategies (see also Hacking, 1992; Elgin, 2017, chs. 8 and 12; Buekens and Smit, 2018). Both rely on contingent and deliberate *choices* regarding what Feyerabend called “styles” of inquiry, where a style is roughly an epistemic stance, perspective, or approach one adopts. Chiara Ambrosio notes that

[t]he choice of a style for Feyerabend is a social act in the sciences as much as it is in the arts, and the analogy between the two fields aims precisely at fleshing out how criteria of truth, reality, success and verification are *internal* to the particular style that communities decide to adopt at a certain time in history (2021, p. 24 emphasis added).

As read here, Feyerabend would thus agree with SESS_int_ that there are no community-extrinsic constraints on or determinants of either scientific or artistic norms (even if community-extrinsic factors might be taken into consideration). Norms for theory-selection and artwork-selection are determined by choices internal to whatever community makes these decisions.

The problem is that Feyerabend only compares science to mimetic art and therefore overlooks that abstract art (which makes up a significant proportion of modern art) is, by definition, unconcerned with imitation or representation. Conversely, even the most *avante garde* scientific theories must maintain contact with the external physical world for them to be scientific in the first place. Writing about whether art and science are analogous, Kuhn likewise notes that, although there is an aesthetic component to both disciplines,

in the arts, the aesthetic is itself the goal of the work. In the sciences it is, at best, again a tool: a criterion of choice between theories which are in other respects comparable, or a guide to the imagination seeking a key to the solution of an intractable technical puzzle. Only if it unlocks the puzzle, only if the scientist’s aesthetic turns out to coincide with nature’s, does it play a role in the development of science (1977a, p. 342; see also Pinto de Oliveira, 2017).

Thus, although aesthetics can play a role in theory-selection, “coincidence with nature” is primary. Even if some scientific enterprises’ goal is pragmatic rather than representational, observations of and/or predictions about the external physical world still play the determining role in developing and selecting theories.

Wray has discussed Feyerabend’s *principle of proliferation*, which states that

there are circumstances when it is admissible to introduce, elaborate and defend *ad hoc* hypotheses [or theories], which contradict well-established and generally accepted experimental results, or hypotheses [or theories] whose content is smaller than the content of the existing and empirically adequate alternative (Feyerabend in Wray 2021, p. 74).

This appears to be a dismissal on Feyerabend’s part of the testability norm. Feyerabend, says Wray, “wants us to see that when choosing theories, scientists should not be constrained by ‘the facts’. The facts, after all, could be contaminated” (2021, p. 75). Indeed; but the principle of proliferation does not encourage scientists to commit to theories that are detached from empirical testing. Instead, Feyerabend thinks that comparing and selecting theories “starts from a certain aim – to obtain *testable* knowledge…” (1981, p. 110 emphasis added). For Feyerabend, the circumstances when “it is admissible to introduce, elaborate and defend ad hoc hypotheses [or theories]” is when they improve the “*testability* of our knowledge” (Feyerabend in Wray 2021, p. 74 emphases added). That is, when they aid scientists in developing theories that are more empirically adequate (to use van Fraassen’s term) than their predecessors. Feyerabend rejects the idea that successful scientific theories correspond to ‘the facts’, but he does think that they coincide empirically with observable phenomena.

Thus, Feyerabend’s putative rejection of the testability norm does not introduce *anything goes* when it comes to norms for theory-selection.^13^ Instead, the principle of proliferation suggests a *short-term* strategy – a kind of methodological ‘opportunism’ – that aids scientists’ *long-term* search for theories that conform to the testability norm (see notably Feyerabend, 1975; see also Shaw, 2017; van der Merwe forthcoming-a). Even if science can be ‘anarchistic’, there is at least one norm – the testability norm – that is not open to radical disruption.

## Conclusion

In this paper, I identified two versions of SESS: SESS_int_ and SESS_ext_. I then argued that, were SESS_int_ correct, the theory-selection practices that determine theory-success in science should be faddish. They should be faddish in the way that the artwork-selection practices that determine artwork-success in art are. Science is however not faddish in this way. Instead, norms for theory-selection have remained largely stable over the last three hundred years, and there is no reason to expect an upheaval of these norms any time soon. I then argued that one norm for theory-selection – the testability norm – explains this stability. The testability norm is definitive of scientific inquiry, and it grounds science’s theory-selection practices and therefore theory-success in the external physical world.

According to SESS_int_, psycho-social factors are the determining explanans for theory-selection. My argument suggests that SESS_ext_ – whether expressed in realist or van Fraassian terms – offers a more plausible explanation. SESS_ext_ appeals to the external physical world (whether observable or unobservable) as the proper explanans for theory-selection. The external physical world is thus the ultimate, rather than proximate, explanation for theory-success in science. There is an explanatory chain that runs from the external physical world to theory-selection to theory-success. Wray’s internalist SESS_int_ only tells half the story since it does not adequately explain the theory-selection practices that explain theory-success. In contrast, SESS_ext_’s externalist approach explains both theory-selection and therefore theory-success. Scientific anti-realists who subscribe to SESS can then explain the success of science by appealing to the external physical world.

## Electronic Supplementary Material

Below is the link to the electronic supplementary material.

Supplementary Material 1

## Footnotes

1 Those involved in the scientific realism/anti-realism debate mostly take ‘science’ to denote the natural sciences. I will follow that convention here.

2 I will use the terms ‘metaphor’ and ‘analogy’ interchangeably.

3 It was suggested during the review process that there may be alternative ways to interpret Wray’s view. Obviously, it is trivially true that any position can be interpreted in different ways. That said, I hope it will become clear through the rest of the paper why my interpretation is the most plausible.

4 Seungbae Park (2014) argues that it is dilemmic for Wray to hold to both SESS and PMI because PMI implies that Darwin’s theory of evolution is false.

5 Realists (e.g. Kitcher, 1993, p. 156; Leplin, 1997, pp. 6–9; Ladyman, 1998, p. 417; Psillos, 1999, pp. 96–97) sometimes state that SESS is unsatisfactory because a genuine explanation for theory-success will tell us something about what makes theories *themselves* successful (see also Stanford, 2020). However, if anti-realists like Wray are concerned with why we have successful theories *at all*, then realists and anti-realists may be taking past each other here.

6 According to Wray, SESS also explains why two competing theories can both be successful. Both successful (yet probably false) theories will be accepted by some scientists (depending on their interests, goals etc.).

7 Standardly, ‘determination’ denotes a strong (even metaphysical or necessary) one-directional relationship (see Wilson 2021 for detail).

9 Van Fraassen’s commitment to observability has been criticised at length (see notably Hacking, 1981 and Rosen, 1994). Those who consider the notion of observability to be problematic can readily substitute the term ‘instrumental detection’ (where the human eye is a kind of instrument).

10 A Kantian might say that the testability norm is a necessary condition for the very possibility of scientific inquiry.

11 By ‘art’, I am referring specifically to institutionalised fine art – e.g. painting, sculpture, music, and performance arts – rather than more ‘practical’ arts, such as gastronomy, satorialism, or craftwork.

12 Even if string theory, for example, which does not (yet) make testable predictions, is a science rather than mathematics, string theorists still hold to something like the testability norm. They are aware that their theory should make testable predictions. It must do so if it is to outcompete rival fundamental physical theories, and thereby achieve iconic scientific status (see Smolin, 2006; Hossenfelder, 2018).

13 Jamie Shaw (2017) mines numerous quotes where Feyerabend insists that his infamous advocation of ‘anything goes’ was never intended to be a positive prescription for how to approach scientific inquiry (see also Farrell, 2003).
