# Supplementary material for: Grounding the Selectionist Explanation for the Success of Science in the External Physical World
Source: Found Sci. 2023 Mar 13;29(4):885–904. doi: 10.1007/s10699-023-09907-y (PMC11568972; doi:10.1007/s10699-023-09907-y)
Supplement: Supplementary file 1 — Supplementary Material 1 [file 10699_2023_9907_MOESM1_ESM.docx]

**Title**

Grounding the Selectionist Explanation for the Success of Science in the External Physical World

**Author:** Ragnar van der Merwe

**Email:** [ragnarvdm@gmail.com](mailto:ragnarvdm@gmail.com)

**Orcid id:** 0000-0003-1038-758X

**Affiliations:** University of Johannesburg, Department of Philosophy, Faculty of Humanities, Kingsway Campus, Corner Kingsway and University Road, Auckland Park, Johannesburg, 2000.

**Abstract**

I identify two versions of the scientific anti-realist’s selectionist explanation for the success of science: Bas van Fraassen’s original and K. Brad Wray’s newer interpretation. In Wray’s version, psycho-social factors internal to the scientific community – *viz*. scientists’ interests, goals, and preferences – explain the theory-selection practices that explain theory-success. I argue that, if Wray’s version were correct, then science should resemble art. In art, the artwork-selection practices that explain artwork-success appear faddish, i.e. prone to radical changes over time. Theory-selection practices that explain theory-success in science are however not faddish; they are mostly stable, i.e. long-lived, and consistent over time. This is because scientists (explicitly or implicitly) subscribe to what I will call the testability norm: scientific theories must make falsifiable claims about the external physical world. The testability norm and not psycho-sociology explains the theory-selection practices that explain theory-success in science. Contra Wray, scientific anti-realists can thus maintain that the external physical world (as entailed in the testability norm) explains theory-success.

**Key words**

Bas van Fraassen; K. Brad Wray; scientific realism; scientific anti-realism; scientific explanation; scientific revolutions

**Declarations**

**Funding**

John Templeton Foundation Project ID: 61408, *Increasing Complexity: The First Rule of Evolution?*

**Conflicts of interest/Competing interests**

The Author declares that there is no conflict of interest
